# *Toxoplasma gondii* activates a Syk-CARD9-NF-κB signaling axis and gasdermin D-independent release of IL-1β during infection of primary human monocytes

**DOI:** 10.1371/journal.ppat.1007923

**Published:** 2019-08-26

**Authors:** William J. Pandori, Tatiane S. Lima, Sharmila Mallya, Tiffany H. Kao, Lanny Gov, Melissa B. Lodoen

**Affiliations:** Department of Molecular Biology & Biochemistry and the Institute for Immunology, University of California, Irvine, California, United States of America; University of New Mexico, UNITED STATES

## Abstract

IL-1β is a potent pro-inflammatory cytokine that promotes immunity and host defense, and its dysregulation is associated with immune pathology. *Toxoplasma gondii* infection of myeloid cells triggers the production and release of IL-1β; however, the mechanisms regulating this pathway, particularly in human immune cells, are incompletely understood. We have identified a novel pathway of *T*. *gondii* induction of IL-1β via a Syk-CARD9-NF-κB signaling axis in primary human peripheral blood monocytes. Syk was rapidly phosphorylated during *T*. *gondii* infection of primary monocytes, and inhibiting Syk with the pharmacological inhibitors R406 or entospletinib, or genetic ablation of Syk in THP-1 cells, reduced IL-1β release. Inhibition of Syk in primary cells or deletion of Syk in THP-1 cells decreased parasite-induced *IL-1β* transcripts and the production of pro-IL-1β. Furthermore, inhibition of PKCδ, CARD9/MALT-1 and IKK reduced p65 phosphorylation and pro-IL-1β production in *T*. *gondii*-infected primary monocytes, and genetic knockout of PKCδ or CARD9 in THP-1 cells also reduced pro-IL-1β protein levels and IL-1β release during *T*. *gondii* infection, indicating that Syk functions upstream of this NF-κB-dependent signaling pathway for IL-1β transcriptional activation. IL-1β release from *T*. *gondii*-infected primary human monocytes required the NLRP3-caspase-1 inflammasome, but interestingly, was independent of gasdermin D (GSDMD) cleavage and pyroptosis. Moreover, GSDMD knockout THP-1 cells released comparable amounts of IL-1β to wild-type THP-1 cells after *T*. *gondii* infection. Taken together, our data indicate that *T*. *gondii* induces a Syk-CARD9/MALT-1-NF-κB signaling pathway and activation of the NLRP3 inflammasome for the release of IL-1β in a cell death- and GSDMD-independent manner. This research expands our understanding of the molecular basis for human innate immune regulation of inflammation and host defense during parasite infection.

## Author summary

IL-1β is a proinflammatory cytokine that contributes to host defense against infection and is also associated with autoimmune and inflammatory diseases. Our prior research has demonstrated that the intracellular parasite *Toxoplasma gondii* induces IL-1β release from primary human monocytes during infection. Here we report the novel finding that within minutes of infection, *T*. *gondii* activates a spleen tyrosine kinase (Syk), PKCδ, CARD9/MALT-1, and NF-κB signaling pathway that is critical for the production of IL-1β in primary human monocytes. We have also investigated the mechanism of IL-1β release from monocytes. Interestingly, although IL-1β can be released during pyroptotic cell death, which is driven by gasdermin family proteins such as gasdermin D (GSDMD), we have found that *T*. *gondii* triggers the release of IL-1β from viable cells, independent of GSDMD, thereby preserving the parasite’s intracellular niche. These studies provide mechanistic insight into the regulation of inflammation and host defense against parasite infection by human innate immune cells.

## Introduction

*Toxoplasma gondii* is an obligate intracellular foodborne parasite capable of infecting and replicating in any nucleated cell of its infected hosts [1]. Global estimates suggest that as much as a third of the world population is chronically infected with this parasite and that over thirty million people become ill from *T*. *gondii* infections each year [2,3]. While a robust immune response typically controls the infection, *T*. *gondii* poses severe health risks to immunocompromised individuals and to the developing fetus during congenital disease [4,5]. In particular, CD4^+^ and CD8^+^ T cells and the production of IFN-γ are required for protection against *T*. *gondii* infection [6,7]. Innate immune cells also contribute significantly to host defense against *T*. *gondii* through the production of IL-12 and cell intrinsic mechanisms of host defense [8]. Monocytes, in particular, are among the first cells recruited to sites of *T*. *gondii* infection and are critical for parasite control during both the acute and chronic stages of disease [9–14].

IL-1β is a potent pro-inflammatory cytokine that is induced by infection and injury and coordinates both the innate and adaptive immune responses [15]. Uncontrolled production of IL-1β has been implicated in the pathogenesis of a variety of diseases such as atherosclerosis, arthritis, diabetes, inflammatory bowel disease, and Alzheimer’s disease [16,17], indicating that IL-1β production and release must be tightly controlled to maintain healthy immune function, during both homeostasis and infection.

Myeloid cells, such as macrophages and monocytes, are major producers of IL-1β during infection or injury. Macrophages regulate IL-1β via a two-signal model. The first signal (Signal 1) is typically induced by Toll-like receptor (TLR) engagement and MyD88 signaling that results in NF-κB nuclear translocation and *IL-1β* transcription [18]. IL-1β is then translated as a biologically inactive pro-protein that cannot bind to the IL-1 receptor until it is cleaved into the mature form by a protease, such as caspase-1. The second signal (Signal 2) activates a multiprotein complex called the inflammasome, of which at least five have been described, which leads to caspase-1 activation and IL-1β cleavage and release [19]. Interestingly, inflammasomes are differentially regulated in macrophages and monocytes [20], and even in human and mouse monocytes: human monocytes activate the inflammasome and release IL-1β in response to LPS alone, using a “one-step” pathway, whereas mouse monocytes stimulated with LPS require an additional Signal 2 for IL-1β cleavage and release [21]. These differences in response to stimulation may reflect unique species- and cell-specific strategies for the regulation and induction of inflammation. Inflammasome activation and IL-1β production are also differentially regulated depending on the nature of the stimulus, which can be as diverse as pathogen infection, microbial products, or sterile inducers of inflammation.

Unlike most cytokines, IL-1β does not possess a signal peptide or traffic through the standard secretory pathway [22]. Instead the best-characterized mechanism of IL-1β release from myeloid cells is through an inflammatory form of cell death known as pyroptosis [23]. Activation of the noncanonical or canonical NLRP3 inflammasome induces pyroptosis through caspase-11- or caspase-1-mediated cleavage of gasdermin D (GSDMD) [24,25]. The cleaved N-terminal domain of GSDMD then inserts into the plasma membrane, where it forms pores through which IL-1β can pass. These pores allow for an influx of extracellular fluid, cell swelling, and eventually pyroptosis, which can release any remaining IL-1β into the extracellular space [26–28]. Recent work has shown GSDMD-dependent pore formation can also mediate IL-1β release from viable “hyperactivated” cells [29], suggesting that GSDMD could serve as a critical mechanistic unifier for the release of IL-1β from both pyroptotic and viable, non-pyroptotic cells.

IL-1β production contributes to host control of *T*. *gondii* infection [30–33], and we have previously shown that *T*. *gondii* infection of primary human monocytes induces the production of IL-1β transcripts and activation of the NLRP3 inflammasome [34,35]. However, *T*. *gondii* infection does not activate any known human TLRs, and the signaling pathways involved in TLR-independent IL-1β production during infection, particularly in human cells, remain poorly defined. In the current study, we demonstrate that primary human monocytes infected with *T*. *gondii* produced IL-1β through a Syk-PKCδ-CARD9/MALT-1-NF-κB signaling pathway and activated the NLRP3 inflammasome for IL-1β release from viable cells in a GSDMD-independent manner. Moreover, we have defined differences in the role of Syk in *T*. *gondii*-infected compared to LPS-stimulated primary human monocytes: during *T*. *gondii* infection, Syk was critical for pro-IL-1β synthesis, whereas in LPS-stimulated monocytes, Syk predominantly mediated IL-1β release. These studies detailing the activation and regulation of the IL-1β pathway during infection and in response to microbial products further our understanding of how primary human immune cells regulate inflammation when activated by diverse stimuli.

## Results

### *T*. *gondii* infection activates the canonical NLRP3 inflammasome and induces the release of bioactive IL-1 from primary human monocytes

**Fig 1:**
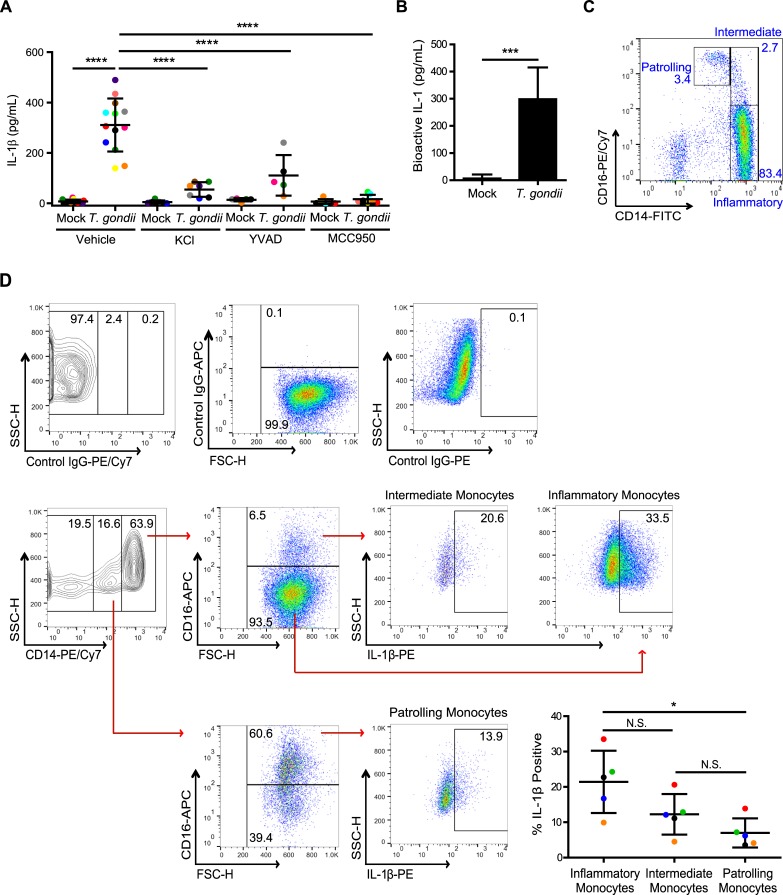
*T*. *gondii*-infected human monocytes release bioactive IL-1β through the NLRP3 inflammasome. (**A**) Primary human peripheral blood monocytes were pretreated with 50 mM potassium chloride, 50 μM YVAD, 2 μM MCC950, or vehicle control for 40 min. Cells were then mock treated or infected with *T*. *gondii* for 4 h, and the levels of IL-1β in the supernatant were measured by ELISA. Each dot represents the response of an individual donor’s cells. (**B**) Primary monocytes were mock treated or infected with *T*. *gondii* for 4 h, and bioactive IL-1 in the supernatant was detected using HEK-Blue IL-1R reporter cells. (**C**) Primary monocytes were analyzed by flow cytometry, and the three monocyte subsets (patrolling, intermediate and inflammatory monocytes) were observed based on CD14 and CD16 expression. (**D**) Primary monocytes were infected with *T*. *gondii* for 4 h, fixed, permeabilized, stained with control Ig or anti-IL-1β, anti-CD14 and anti-CD16 antibodies, and analyzed by flow cytometry. The representative gating strategy is shown (left). The percentage of IL-1β positive cells was measured in the three subsets of monocytes (right). Data in (A) reflect combined results of 5–13 experiments with independent donors. For (B), combined data from 5 donors are shown. In (C), a representative plot from over 70 monocyte isolation experiments is shown. In (D), combined results from 5 experiments with different donors are shown. Values are expressed as the mean ± SD, *P<0.05, ***P<0.001, ****P<0.0001 (one-way ANOVA followed by a Tukey post-test in A, B, and D).

To investigate IL-1β production and release from primary human monocytes during *T*. *gondii* infection, we isolated monocytes from healthy blood donors as previously described (S1 Fig and [34]) and immediately infected the cells *in vitro* with GFP-expressing *T*. *gondii* or treated them with an equal volume of culture medium (mock). At 4 hours post-infection (hpi), IL-1β released into the supernatant was detected by ELISA, and the response of cells from individual human donors was compared (each dot represents a unique donor) (Fig 1A). The pretreatment of primary monocytes with MCC950, an NLRP3 inhibitor; YVAD, a caspase-1 inhibitor; or KCl, which prevents K^+^ efflux and inhibits activation of the canonical NLRP3 inflammasome [36], all resulted in a significant decrease in IL-1β release from infected primary human monocytes from multiple independent donors (Fig 1A). Notably, none of these treatments or inhibitors affected the efficiency of infection, as determined by the percent of GFP^+^ (infected) cells in the culture (S2A Fig) or the ability of the parasite to replicate in and lyse human foreskin fibroblasts (HFFs), as determined by plaque assays (S2B Fig). The transfer of supernatants from *T*. *gondii-*infected, but not mock-treated monocytes to the HEK-Blue reporter cell line resulted in reporter cell activation, indicating the release of functional IL-1 from the infected primary monocytes (Fig 1B).

There are three subsets of peripheral blood monocytes that have been described in humans, which are defined by their relative expression of CD14 and CD16 [37] (Fig 1C). Recent publications have shown that the CD14^+^CD16^-^ inflammatory subset of monocytes is associated with increased and chronic inflammation and the development of arthritis [38,39]. Using intracellular cytokine staining (ICCS) to compare IL-1β production in each subset, we found that *T*. *gondii* infection stimulated all three subsets to produce IL-1β by 4 hpi (Fig 1D). We also observed that in each of the five donors analyzed, the CD14^+^CD16^-^ inflammatory monocytes exhibited the highest percentage of IL-1β^+^ cells (Fig 1D). Collectively, these data demonstrate that *T*. *gondii* triggers the production of IL-1β in all subsets of primary peripheral blood human monocytes and activates the canonical NLRP3 inflammasome for the release of bioactive IL-1 by 4 hpi.

### IL-1β release from primary human monocytes is dependent on Syk

**Fig 2:**
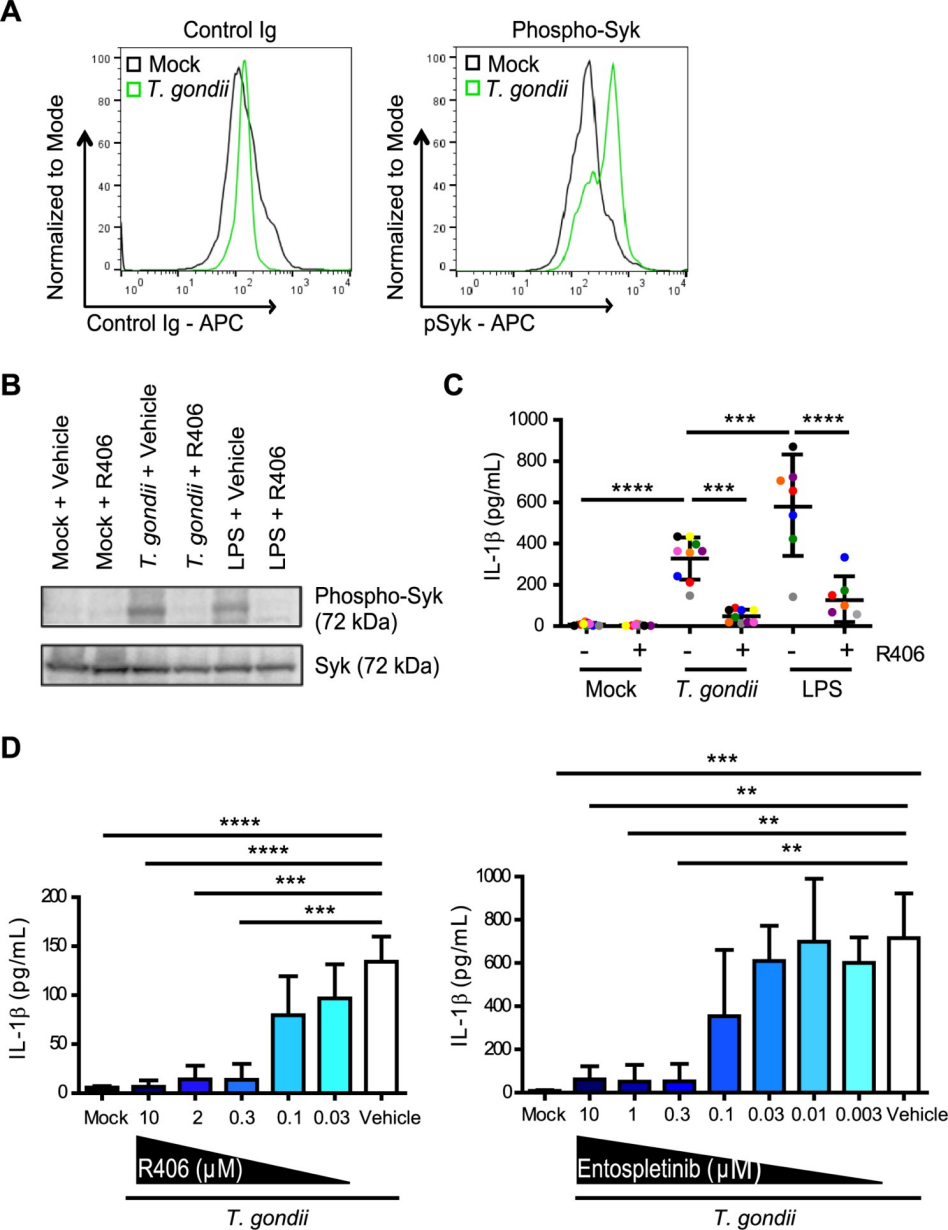
Syk is activated during *T*. *gondii* infection of primary human monocytes. (**A**) Primary human monocytes were mock treated or infected with *T*. *gondii*. After 30 min, cells were permeabilized, stained with control Ig or anti-phospho-Syk (Tyr525/526) antibody, and flow cytometry was performed. (**B**) Primary monocytes were treated with 2 μM R406 or vehicle control and then mock treated, infected with *T*. *gondii*, or stimulated with LPS (100 ng/ml) for 30 min. Total Syk and phospho-Syk (Tyr525/526) in the cell lysate were visualized by Western blotting. (**C**) Primary monocytes were pretreated with 2 μM R406 or vehicle control for 40 min and then mock treated, infected with *T*. *gondii*, or stimulated with LPS (100 ng/ml) for 4 h, and the levels of IL-1β in the supernatant were measured by ELISA. (**D**) Primary monocytes were pretreated with different concentrations of R406 (0.03–10 μM), entospletinib (0.003–10 μM), or vehicle control for 40 min. Monocytes were then mock treated or infected with *T*. *gondii* for 4 h, and the levels of IL-1β in the supernatant were measured by ELISA. Representative plots of 5 experiments are shown in (A). In (B), a representative Western blot from 4 experiments is shown. Data in (C) and (D) reflect combined results of 7 and 3 experiments with independent donors, respectively. Values are expressed as the mean ± SD, **P<0.01, ***P<0.001, ****P<0.0001 (one-way ANOVA followed by a Tukey post-test in panel C and a Dunnett post-test in panel D).

Syk is a tyrosine kinase that is expressed in hematopoietic cells and is involved in NLRP3 activation during fungal infection, viral infection, and in response to LPS stimulation [40–43]. However, the role of Syk in IL-1β regulation during parasite infection is unknown. Interestingly, rapid phosphorylation of Syk at tyrosine 525/526, which is an indicator of activation, was detected by phospho-flow cytometry (Fig 2A) and Western blotting of lysates (Fig 2B) from monocytes that were infected with *T*. *gondii* or treated with LPS, as a positive control. The phosphorylation of Syk was reduced in the presence of the Syk-specific inhibitor R406 (Fig 2B). To investigate a potential role for Syk in IL-1β release during *T*. *gondii* infection, primary human monocytes were pre-treated with the Syk inhibitor R406 or a vehicle control, and either infected with *T*. *gondii* or treated with LPS. LPS stimulation induced significantly more IL-1β release than *T*. *gondii* infection, but R406 treatment significantly reduced IL-1β release from primary human monocytes treated with either stimulus at the 4 hour time-point (Fig 2C). Titration of R406 revealed a dose-dependent effect of the Syk inhibitor (Fig 2D). Importantly, pretreatment of monocytes with the R406 inhibitor did not reduce the infection efficiency of the parasite or the GFP median fluorescence intensity (MFI) of monocytes infected with GFP-expressing parasites at either 4 hpi or 16 hpi (S3A Fig). In addition, R406 did not decrease the ability of *T*. *gondii* to grow in and lyse HFFs, as measured by plaque assays (S3B Fig), or the viability of monocytes (S3C Fig). Furthermore, an independent Syk inhibitor, entospletinib, which is currently in use in clinical trials for leukemia [44], also reduced IL-1β release from *T*. *gondii*-infected monocytes in a dose-dependent manner (Fig 2D), without affecting parasite infection efficiency (S2A Fig) or viability (S2B Fig). Collectively, these data indicate on-target effects of the Syk inhibitors and demonstrate that *T*. *gondii* infection induces IL-1β release from primary human monocytes in a Syk-dependent manner.

### Syk is required for IL-1β transcript production in *T*. *gondii*-infected primary human monocytes

**Fig 3:**
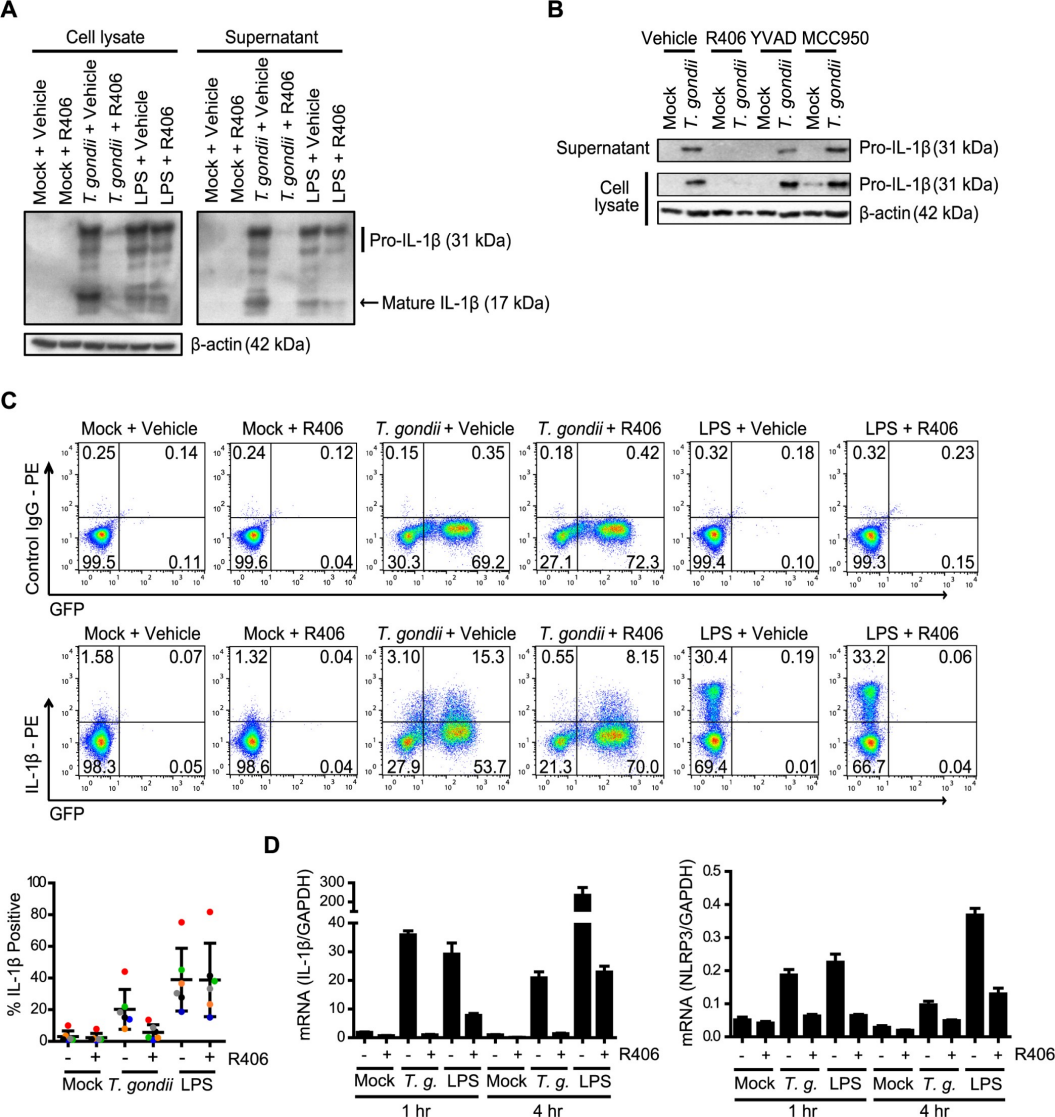
Syk is required for IL-1β production in *T*. *gondii*-infected human monocytes. (**A**) Primary human monocytes were pretreated with 2 μM R406 or vehicle control for 40 min and then mock treated, infected with *T*. *gondii*, or stimulated with LPS (100 ng/ml) for 4 h. Pro- and mature IL-1β in the cell lysate and supernatant, and β-actin in the lysate were visualized by Western blotting. (**B**) Primary monocytes were pretreated with 2 μM R406, 50 μM YVAD, 2 μM MCC950, or vehicle control for 40 min. Cells were then mock treated or infected with *T*. *gondii* for 4 h, and pro-IL-1β in the cell lysate and supernatant was visualized by Western blotting. (**C**) Cells were pretreated with 2 μM R406 or vehicle control for 40 min and then mock treated, infected with *T*. *gondii*, or stimulated with LPS (100 ng/ml). After 4 h, cells were fixed, permeabilized, stained with control Ig or anti-IL-1β, and analyzed by flow cytometry. The percentage of IL-1β positive cells in each condition was plotted. (**D**) Monocytes were treated as in (A), qPCR was performed with primers specific for *IL-1β* and *NLRP3*, and transcript levels relative to those of *GAPDH* are graphed. Representative Western blots from 4 (A and B) experiments are shown. For the graph in (C), data show combined results from 6 experiments with independent donors, and representative FACS plots are shown. In (D), representative data from 4 experiments are shown. Values are expressed as the mean ± SD.

Syk has been proposed to play two roles in the regulation of IL-1β production in other models: inducing NF-κB activation and IL-1β transcription via the PKCδ-CARD9/MALT-1-NF-κB pathway or indirectly activating NLRP3 inflammasome assembly via ASC phosphorylation and oligomerization [45,46]. In the lysates and supernatants of primary human monocytes pretreated with the Syk inhibitor R406, we observed a marked reduction in both pro- and mature IL-1β protein levels compared to infected monocytes treated with the vehicle control (Fig 3A). In contrast, R406 pre-treatment of LPS-stimulated primary monocytes did not reduce the levels of pro-IL-1β protein in the cell lysates (Fig 3A). Notably, the NLRP3 and caspase-1 inhibitors MCC950 and YVAD, respectively, had no effect on pro-IL-1β synthesis or release from primary monocytes, as expected (Fig 3B). ICCS of primary human monocytes infected with *T*. *gondii* or treated with LPS in the presence or absence of R406 indicated that Syk inhibition reduced the percentage of intracellular IL-1β^+^ *T*. *gondii-*infected monocytes but did not decrease the percentage of intracellular IL-1β^+^ monocytes stimulated with LPS (Fig 3C).

To directly examine the effect of Syk inhibition on IL-1β and NLRP3 transcript levels, qPCR was performed on samples from human monocytes infected with *T*. *gondii* or treated with LPS in the presence or absence of R406. These data corroborated the Western blot and ICCS data and demonstrated that R406 decreased *IL-1β* and *NLRP3* transcripts at 1 and 4 hpi in *T*. *gondii*-infected monocytes (Fig 3D). Interestingly, R406 treatment also reduced *IL-1β* and *NLRP3* transcripts in LPS-stimulated monocytes (Fig 3D), despite having little to no effect on pro-IL-1β levels in these cells (Fig 3A). Together these data suggest that Syk signaling is critical for production of *IL-1β* and *NLRP3* transcripts in *T*. *gondii*-infected primary human monocytes, and therefore appears to act in the priming stage of IL-1β production during infection.

### IL-1β synthesis and release are reduced during *T*. *gondii* infection of Syk knockout THP-1 cells

**Fig 4:**
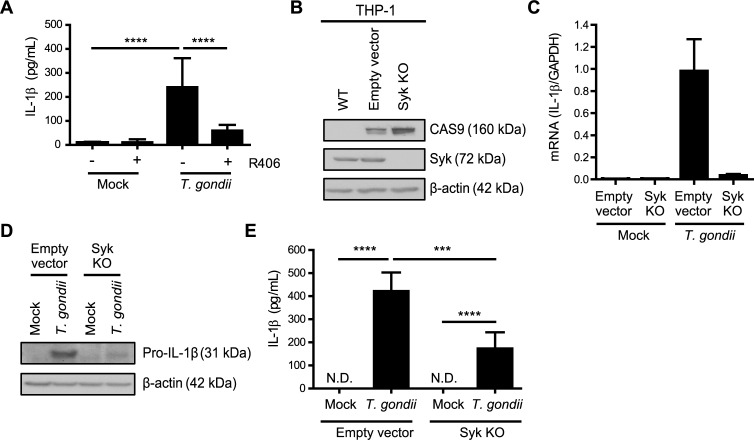
Syk contributes to IL-1β production in *T*. *gondii*-infected monocytic THP-1 cells. (**A**) THP-1 cells were pretreated with 2 μM R406 or vehicle control for 40 min and then mock treated or infected with *T*. *gondii* for 18 h, and the levels of IL-1β in the supernatant were measured by ELISA. (**B**) Lysates from wild-type (parental) THP-1 cells, control Empty Vector THP-1 cells, and Syk KO THP-1 cells were blotted with antibodies to visualize Cas9, Syk, and β-actin. (**C**) Empty Vector THP-1 cells and Syk KO THP-1 cells were mock treated or infected with *T*. *gondii* and qPCR was performed with primers specific for *IL-1β*. Transcript levels relative to those of *GAPDH* are graphed. (**D** and **E**) Empty Vector or Syk KO THP-1 cells were mock treated or infected with *T*. *gondii*, and pro-IL-1β and β-actin in the cell lysate were visualized by Western blotting (**D**) or the levels of IL-1β in the supernatant were measured by ELISA (**E**). In (A) and (E), combined data from 4 experiments are shown. Representative Western blots (B and D) and qPCR (C) from 4 experiments are shown. Values are expressed as the mean ± SD, ***P<0.001, ****P<0.0001 (one-way ANOVA followed by a Tukey post-test in A and E).

Since primary human monocytes cannot be genetically manipulated (or reliably cultured *in vitro* for more than ~24 hours), we examined a role for Syk in the human monocytic cell line THP-1. These cells also release IL-1β in response to *T*. *gondii* infection, but with delayed kinetics compared to primary monocytes [34,35]. Similar to primary monocytes, *T*. *gondii* infection induced Syk phosphorylation in THP-1 cells (S4A Fig), and pre-treatment of THP-1 cells with R406 resulted in the release of significantly less IL-1β than in infected THP-1 cells treated with the vehicle control (Fig 4A). To complement the R406 inhibitor experiments, THP-1 cells were transduced with lentivirus carrying guide RNAs targeting Syk for CRISPR/Cas9-mediated genome editing. As a control, THP-1 cells were transduced with an empty vector (EV) lacking the Syk targeting sequence. Unlike in the wild-type (WT) parental THP-1 cells, Cas9 was detected in the EV control and Syk KO lines, and Syk was absent only from the KO line (Fig 4B). This Syk KO clone harbors mutations in the second SH2 domain, resulting in a frameshift mutation that alters the amino acid sequence of the targeted exon (S4 Fig).

The control EV line and the Syk KO THP-1 cells were infected with *T*. *gondii* and examined for IL-1β production. qPCR analysis revealed reduced levels of *IL-1β* mRNA in the Syk KO THP-1 cells compared to the EV control cells (Fig 4C). Similarly, reduced levels of pro-IL-1β protein were detected in the Syk KO cells compared to the EV control cells during infection (Fig 4D), suggesting that Syk functions upstream of *IL-1β* transcription and pro-IL-1β protein synthesis in THP-1 cells, similar to the effects of the R406 and entospletinib inhibitors in primary human monocytes. Finally, Syk KO THP-1 cells released less IL-1β in the supernatant, as detected by ELISA, than the control EV cells during *T*. *gondii* infection (Fig 4E). Collectively, these data indicate that *T*. *gondii* infection induces IL-1β synthesis and release from both primary human monocytes and THP-1 cells in a Syk-dependent manner.

### *T*. *gondii*-infected monocytes activate a Syk-PKCδ-CARD9/MALT-1-NF-κB signaling pathway for the production and release of IL-1β

**Fig 5:**
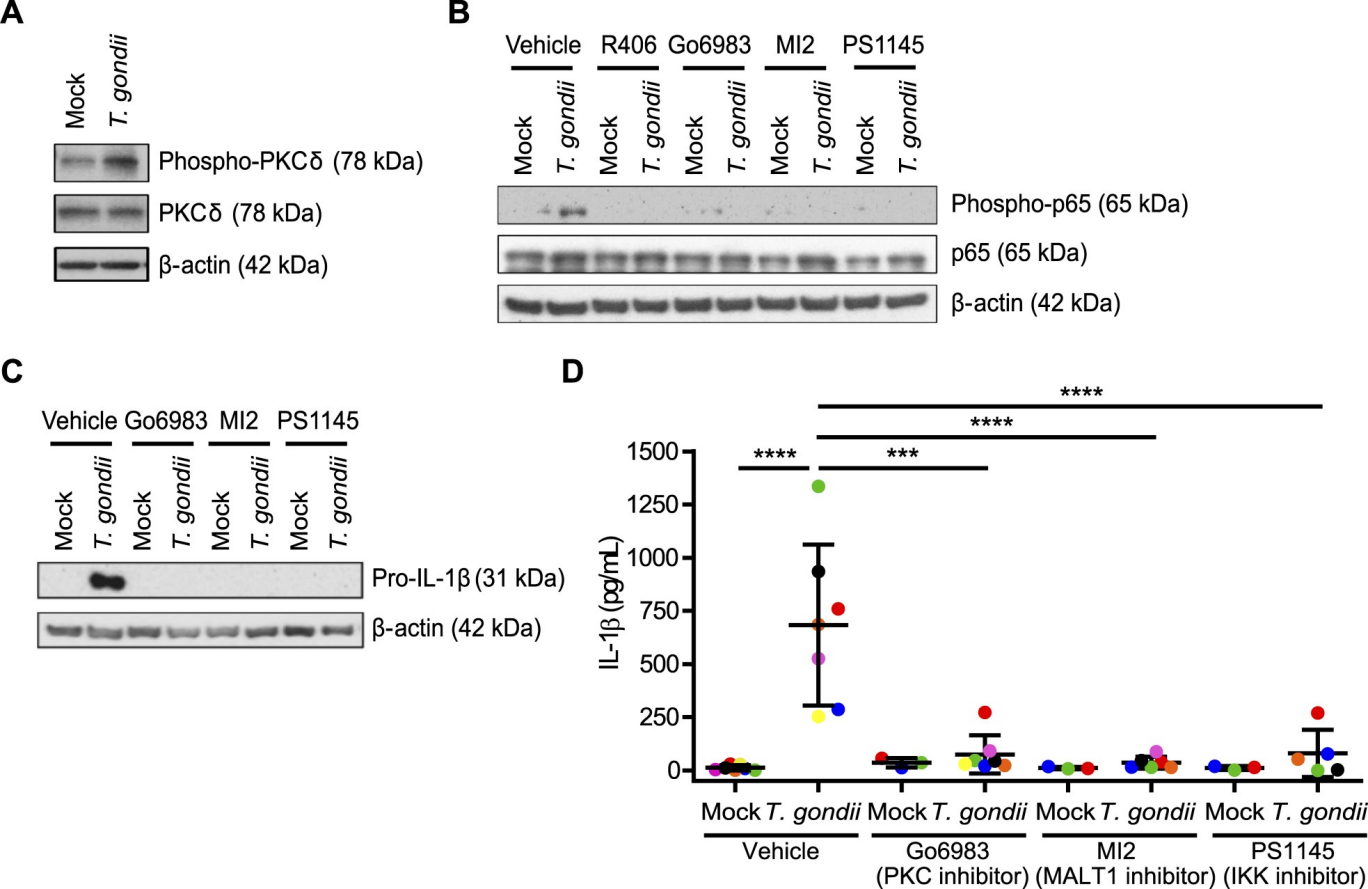
The Syk-PKCδ-CARD9/MALT-1-NF-κB pathway is activated in *T*. *gondii*-infected human monocytes. (**A**) Primary monocytes were mock treated or infected with *T*. *gondii* and the lysates were blotted for total and phospho-PKCδ and for β-actin. (**B**) Primary monocytes were pretreated with 2 μm R406, 300 nM Go6983, 3 μM MI2, 100 nM PS1145, or vehicle control for 40 min then mock treated or infected with *T*. *gondii* for 1 h. Total and phospho-p65 (Ser536) and β-actin in the cell lysate were visualized by Western blotting. (**C**) Pro-IL-1β and β-actin were visualized by Western blotting of lysates from primary monocytes that were mock treated or infected with *T*. *gondii* for 4 hr in the presence of the vehicle control or the indicated inhibitors. (**D**) The levels of IL-1β in the supernatant of mock or *T*. *gondii*-infected primary monocytes in the presence or absence of the indicated inhibitors were measured by ELISA. Data in (D) are combined results of 3–7 experiments with independent donors. Representative Western blots from 3 (A and C) and 4 (B) experiments are shown. Values are expressed as the mean ± SD, ***P<0.001, ****P<0.0001 (one-way ANOVA followed by a Tukey post-test).

While it has been well documented that LPS activates a MyD88-IRAK1/4-TRAF6 pathway resulting in NF-κB nuclear translocation, Syk has been shown to activate an alternative PKCδ-CARD9/MALT-1-NF-κB signaling pathway [46]. To investigate a potential role for this pathway in IL-1β production during *T*. *gondii* infection of human monocytes, we examined the activation of PKCδ and the NF-κB subunit p65 and found that *T*. *gondii* infection induced phosphorylation of both PKCδ and p65 in primary human monocytes (Fig 5A and 5B). Treatment of monocytes with R406; Go6983, a PKC inhibitor which is active against PKCδ; MI2, a MALT-1 and CARD9 complex inhibitor; or PS1145, an IKK inhibitor, prior to *T*. *gondii* infection all significantly reduced p65 phosphorylation induced by *T*. *gondii* infection (Fig 5B), indicating that the inhibitors all targeted a pathway upstream of NF-κB activation. Similar to the Syk inhibitor, the PKCδ, CARD9/MALT-1, and IKK inhibitors all reduced pro-IL-1β protein production in *T*. *gondii*-infected primary human monocytes at 4 hpi (Fig 5C). Consistent with these data, IL-1β release was significantly reduced in primary human monocytes treated with these inhibitors compared to vehicle control-treated monocytes during infection, as determined by ELISA (Fig 5D). Importantly, the inhibitors did not reduce infection efficiency at 4 hpi (S2A Fig) or affect the ability of the parasites to replicate in and lyse HFFs at the concentrations used (S2B Fig). Together, these data suggest that primary human monocytes rely almost completely on signaling through a Syk-PKCδ-CARD9/MALT-1-NF-κB signaling pathway for IL-1β production during *T*. *gondii* infection.

### Knockout of PKCδ and CARD9 in THP-1 cells decreases *T*. *gondii*-induced IL-1β production

**Fig 6:**
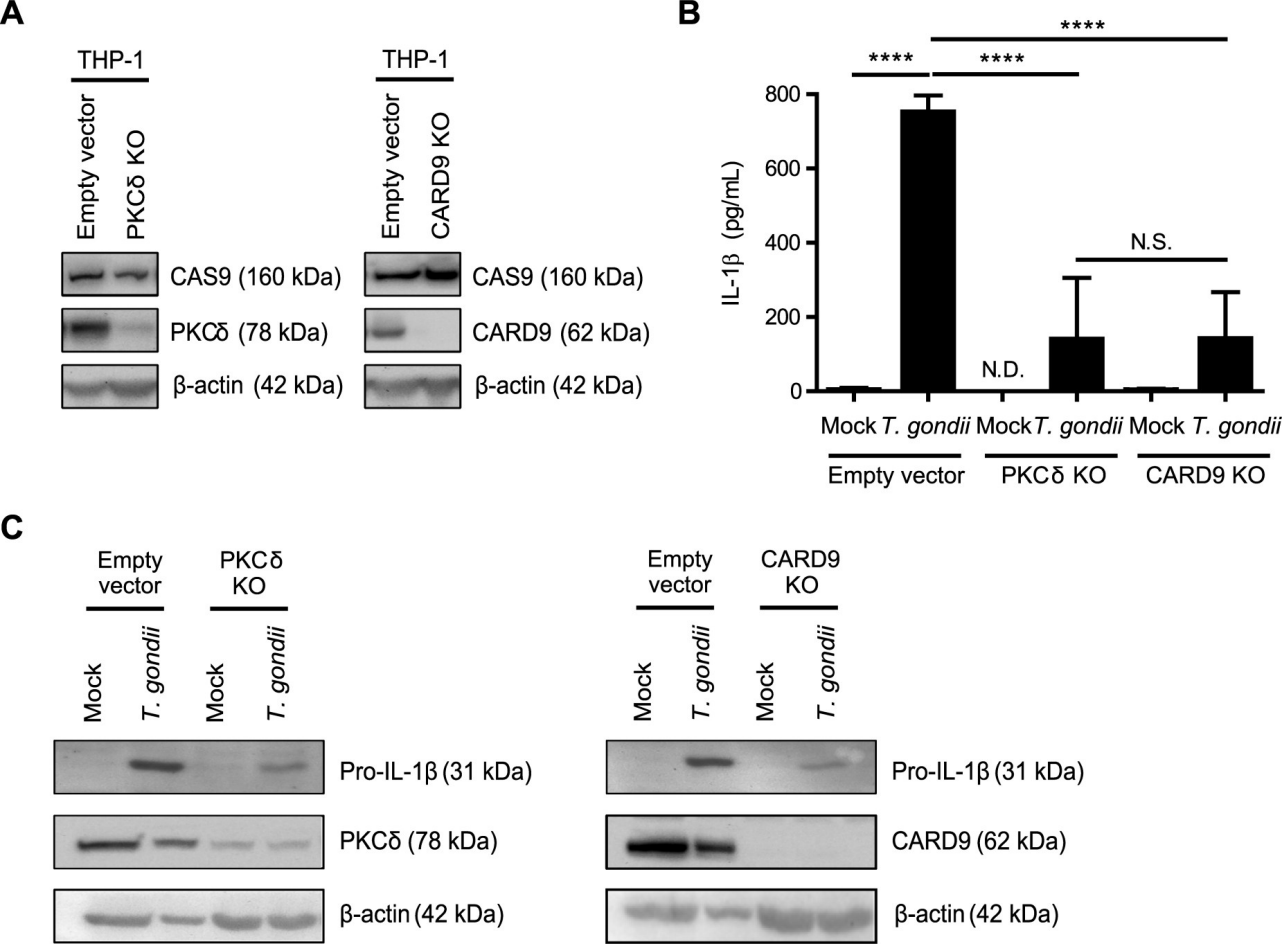
PKCδ and CARD9 contribute to IL-1β production in *T*. *gondii*-infected monocytic THP-1 cells. (**A**) Cell lysates from control Empty Vector THP-1 cells, PKCδ KO, and CARD9 KO THP-1 cells were blotted with antibodies against Cas9, PKCδ, CARD9, and β-actin. (**B**) Empty Vector, PKCδ KO, and CARD9 KO THP-1 cells were mock treated or infected with *T*. *gondii* for 18 h, and the levels of IL-1β in the supernatant were measured by ELISA. (**C**) Samples from the same experiments were also used to visualize pro-IL-1β, β-actin, PKCδ, and CARD9 in the cell lysates by Western blotting. Data in (B) are combined results of 4 experiments. Representative Western blots from 4 (A and C) experiments are shown. Values are expressed as the mean ± SD, ****P<0.0001 (one-way ANOVA followed by a Tukey post-test in panel B).

To investigate roles for PKCδ and CARD9 in IL-1β production during *T*. *gondii* infection using a genetic approach, THP-1 cells were subjected to CRISPR/Cas9-mediated genome editing using guide RNAs targeting each of these two proteins. Cas9 protein was detected in the EV control cells and the KO cells, and PKCδ and CARD9 were absent or severely reduced in each of the respective KO populations (Fig 6A). The faint detection of PKCδ in the PKCδ KO population (Fig 6A and 6C) may reflect the fact that these cells represent a mixed population, rather than a clonal KO line. Infection of either the PKCδ KO or CARD9 KO THP-1 cells with *T*. *gondii* resulted in reduced IL-1β release compared to infection of the EV cells (Fig 6B). In addition, *T*. *gondii*-infected PKCδ KO and CARD9 KO THP-1 cells contained less pro-IL-1β protein in the cell lysates than *T*. *gondii-*infected EV THP-1 cells (Fig 6C). These data indicate that both PKCδ and CARD9 contribute to IL-1β synthesis and release from THP-1 cells, consistent with the results obtained using inhibitors of these proteins in primary human monocytes.

### *T*. *gondii*-infected and LPS-stimulated human monocytes release IL-1β via a mechanism that is independent of cell death and GSDMD

**Fig 7:**
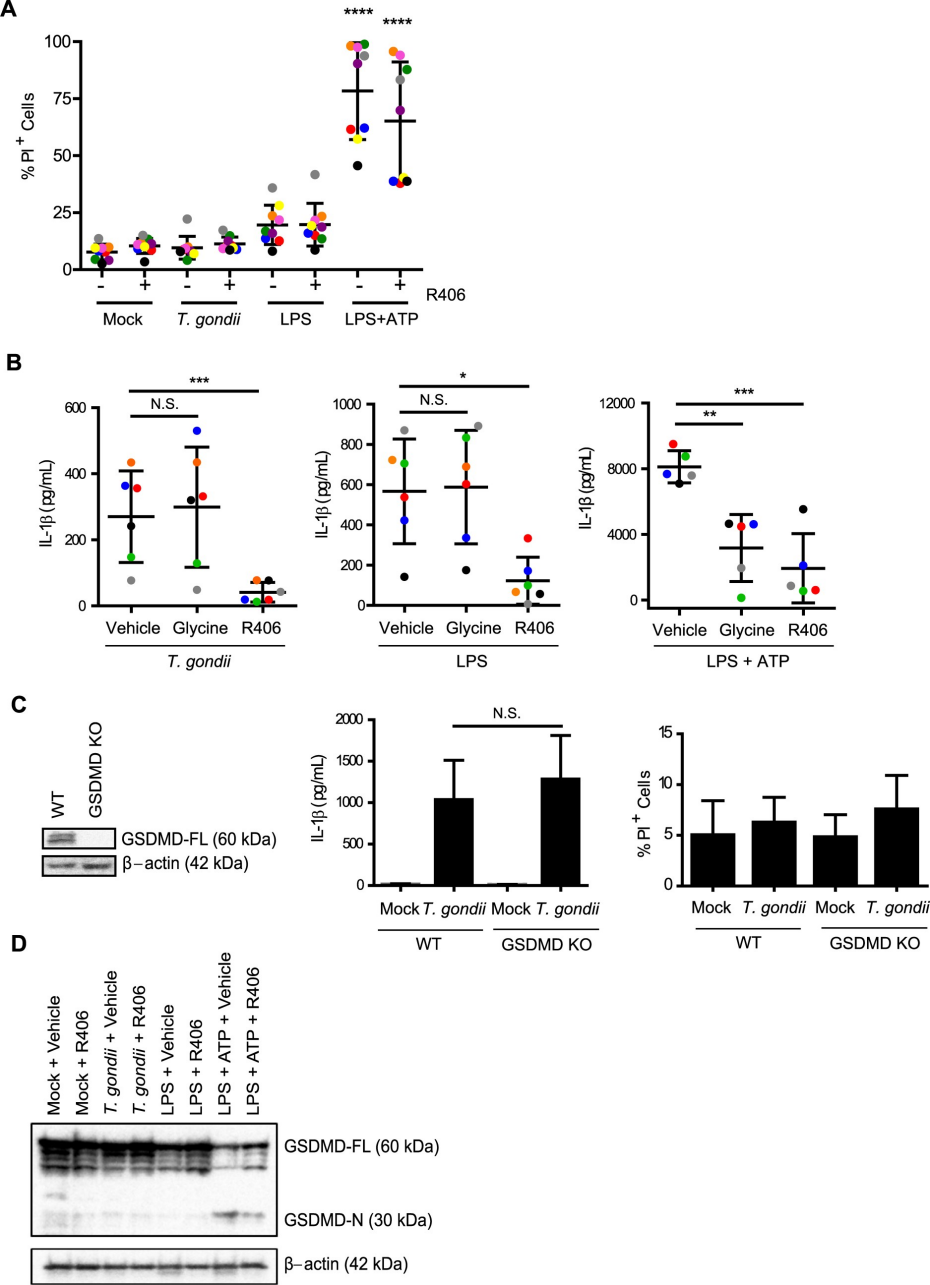
*T*. *gondii*-induced IL-1β release is independent of cell death and gasdermin D (GSDMD). (**A**) Primary human monocytes were pre-treated with 2 μM of the Syk inhibitor R406 or vehicle control (DMSO) for 40 min, mock treated, infected with *T*. *gondii*, stimulated with LPS (100 ng/ml), or stimulated with LPS (100 ng/ml) plus ATP (5 mM) for 4 h (ATP was added for the last 30 minutes of treatment). Cell viability by PI staining was then measured by flow cytometry. (**B**) Primary monocytes were pretreated with 5 mM glycine, 2 μM R406, or vehicle control for 40 min. Cells were then infected with *T*. *gondii*, stimulated with LPS alone or LPS+ATP for 4 h, and the levels of IL-1β in the supernatant were measured by ELISA. (**C**) Lysates from wild type and GSDMD knockout (KO) THP-1 cells were blotted with anti-GSDMD or anti-β-actin antibodies. The WT or GSDMD KO cells were mock treated or infected with *T*. *gondii* for 18 h. The levels of IL-1β in the supernatant were measured by ELISA, and cell viability by PI staining was measured by flow cytometry. (**D**) GSDMD-FL (full-length) and GSDMD-N (N-terminal) protein in the cell lysate were visualized by Western blotting. Data in (A) and (B) reflect combined results of 9 and 5 experiments with independent donors, respectively. For (C), combined data from 6 and 13 experiments is shown for the IL-1β release ELISA and PI staining experiments respectively. In (D) a representative Western blot from 4 experiments is shown. Values are expressed as the mean ± SD, *P<0.05, **P<0.01, ***P<0.001, ****P<0.0001 (one-way ANOVA followed by a Tukey post-test in A, B and C). In (A), the LPS+ATP treated conditions contained a significantly higher percentage of PI^+^ cells than all of the first six conditions, which were not significantly different from each other.

The most well characterized mechanism of IL-1β release due to inflammasome activation is an inflammatory form of cell death, marked by cell membrane pore formation, cell swelling, and lysis, termed pyroptosis [23]. To address a potential role for pyroptosis in IL-1β release during *T*. *gondii* infection, primary human monocytes were mock treated, infected with *T*. *gondii*, or stimulated with LPS or LPS and ATP, as controls. The cells were then stained with propidium iodide (PI), which passes through small pores in the plasma membrane and binds to DNA. At 4 hpi, when bioactive IL-1β release was detected (Fig 1B), the viability of the *T*. *gondii-*infected monocyte population, as measured by the percentage of PI^+^ cells, was indistinguishable from mock-treated cells (Fig 7A), and the addition of the Syk inhibitor R406 did not alter the percentage of PI^+^ cells. In contrast to *T*. *gondii* infection and LPS stimulation, a high level of cell death was detected when cells were treated with the canonical inflammasome activator, LPS and ATP (Fig 7A). Titrating the ATP in LPS-stimulated cells triggered cell death in a dose-dependent manner (S5 Fig). The addition of extracellular glycine, which inhibits ion flux, thereby halting cell swelling and the completion of pyroptosis, reduced IL-1β release from LPS and ATP-stimulated cells but not from LPS-stimulated or *T*. *gondii*-infected cells (Fig 7B). In contrast, Syk inhibition with R406 did reduce IL-1β release in the LPS-stimulated and *T*. *gondii*-infected cells, without affecting cell death (Fig 7A and 7B). Interestingly, the stimulus of LPS and ATP, which induced cell death, led to the release of more than ten times the amount of IL-1β from primary human monocytes than *T*. *gondii* infection or LPS stimulation alone (Fig 7B). These results support the idea that the degree of pyroptosis and IL-1β release during stimulation may relate to the intensity of the stimulus encountered by the cells [47].

Although we did not detect significantly more cell death among *T*. *gondii-*infected monocytes compared to mock-treated cells, we formally tested a role for GSDMD by infecting wild-type (WT) and GSDMD knockout THP-1 cells [48] with *T*. *gondii* and examining IL-1β release by ELISA (Fig 7C). Notably, the GSDMD KO cells were not impaired in their release of IL-1β during *T*. *gondii* infection compared to WT THP-1 cells (Fig 7C), and the viability of these cells was not significantly different than that of mock-treated THP-1 cells at the same time-point (Fig 7C). Furthermore, whereas LPS and ATP stimulation of primary human monocytes led to the cleavage of GSDMD from the full-length 60 kD protein to the N-terminal p30 fragment, neither LPS nor *T*. *gondii* infection resulted in increased GSDMD cleavage at 4 hpi, and Syk inhibition with R406 did not affect GSDMD cleavage in the *T*. *gondii* or LPS conditions (Fig 7D). These data further support the conclusion that LPS and *T*. *gondii* trigger IL-1β release from human monocytes independent of GSDMD cleavage, pore formation, and pyroptosis.

## Discussion

**Fig 8:**
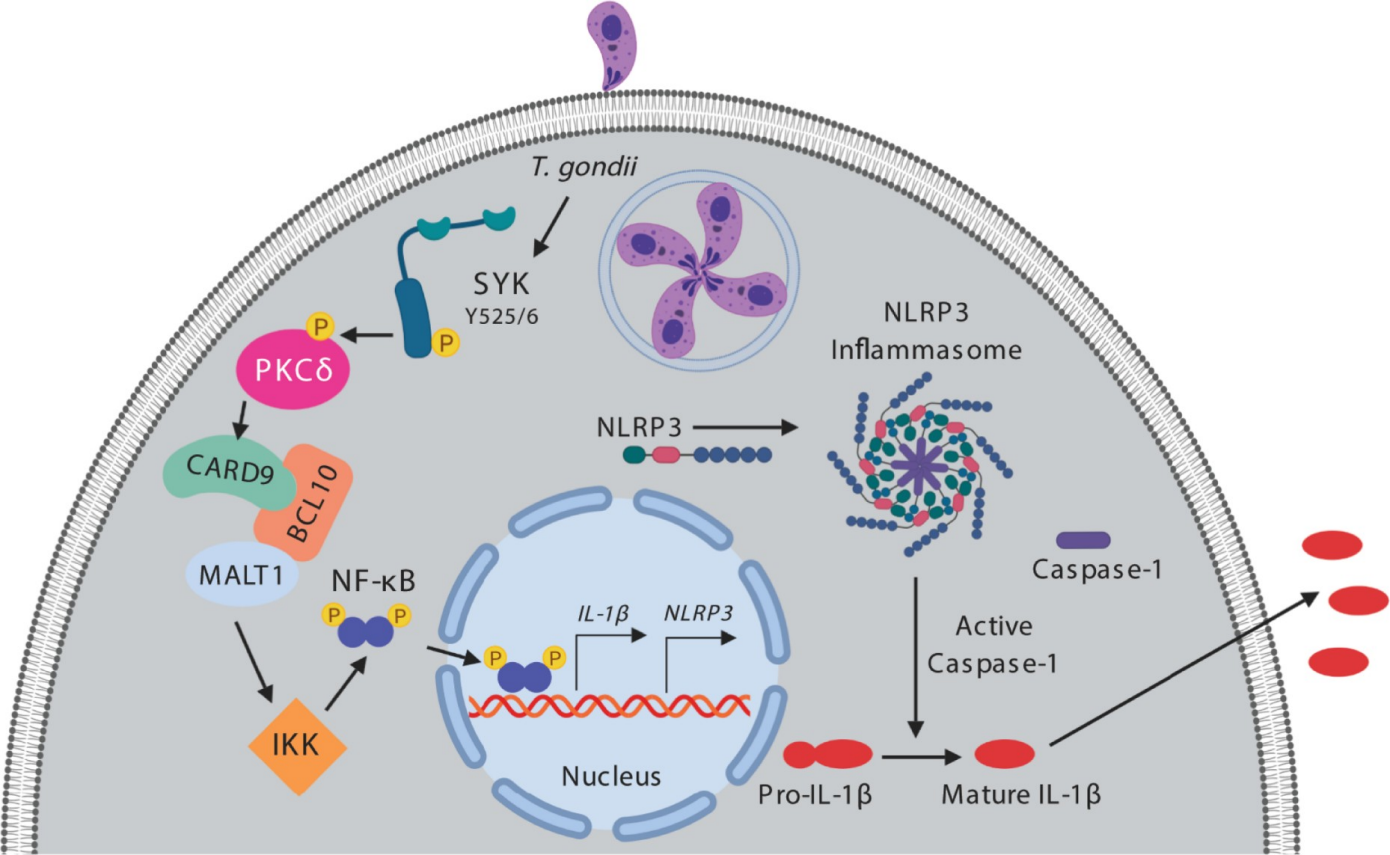
Model for *T*. *gondii*-induced IL-1β production in primary human monocytes. *T*. *gondii* infection induces phosphorylation and activation of Syk at tyrosine 525/6 (Y525/6). Syk activation subsequently leads to the phosphorylation of PKCδ, which activates the CARD9/BCL10/MALT1 complex and downstream IKK. This leads to phosphorylation of the p65 subunit of NF-κB and the translocation of NF-κB into the nucleus where it initiates transcription of *IL-1β* and *NLRP3*. NLRP3 then associates with ASC and caspase-1 to form the canonical NLRP3 inflammasome. Pro-caspase-1 is cleaved into activate caspase-1, which then cleaves pro-IL-1β to mature IL-1β. The mature, bioactive IL-1β exits the cell by an unknown mechanism, independent of pyroptosis, gasdermin D, or pore formation in the plasma membrane.

Syk is a tyrosine kinase expressed in immune cells and is typically activated by receptors or receptor-associated adaptor proteins with cytoplasmic immunoreceptor tyrosine-based activation motifs (ITAMs) [49]. Syk is known to be critical for lymphatic development, inflammatory signaling, and inflammasome activation [50]. We now demonstrate that inhibition of Syk in primary human monocytes and genetic deletion of Syk in monocytic THP-1 cells both significantly reduced *IL-1β* transcripts and pro-IL-1β production during *T*. *gondii* infection, indicating a role for Syk in IL-1β synthesis in parasite-infected cells. Among other signaling pathways, Syk can signal through PKCδ and CARD9 to induce NF-κB activation [46,51], and indeed, inhibitors against PKCδ, CARD9/MALT-1, and IKK, or genetic deletion of PKCδ and CARD9 in THP-1 cells revealed the importance of this pathway in IL-1β synthesis in *T*. *gondii*-infected monocytes, as depicted in Fig 8. Syk also contributed to *NLRP3* transcript induction in response to *T*. *gondii* infection, further supporting a role for Syk in the priming of both IL-1β and NLRP3. In contrast, Syk appeared to be less important for the production of pro-IL-1β in LPS-stimulated primary human monocytes, but rather, seemed to contribute more substantially to IL-1β release from these cells. LPS-stimulated monocytes likely rely more heavily on canonical NF-κB signaling downstream of TLR4 and MyD88 for IL-1β transcription, and on Syk for processing and release of IL-1β. Indeed, it has been shown that Syk can lead to activation of the NLRP3 inflammasome through the indirect phosphorylation of the inflammasome adaptor protein ASC [45,52]. ASC phosphorylation induces its oligomerization, facilitating activation of the NLRP3 inflammasome and caspase-1 [53,54]. Collectively, our data indicate that immune cells responding to pathogens harboring multiple PAMPs or vitaPAMPs will likely regulate IL-1β differently than cells responding to a single PAMP or stimulus. For example, primary human monocytes utilize Syk signaling through ERK1/2 to produce IL-1β when dengue virus is in complex with antibody [43], and our research shows that during *T*. *gondii* infection Syk activates a separate signaling pathway to reach the same net result. Thus, examining the production and processing of IL-1β and other cytokines during infection with various live pathogens may unveil new pathways of regulation that could be critical for enhancing or dampening inflammation during different types of infections.

In mice, *T*. *gondii* is sensed by TLR11/12 recognition of the parasite actin-binding protein profilin [55–57]; interestingly, however, these TLRs are not functional in humans. And although Syk can be activated downstream of TLR4 [41,58], there is no known human TLR that has been shown to recognize *T*. *gondii*, suggesting that *T*. *gondii*-induced Syk signaling occurs via a different receptor. Recent work has shown that *T*. *gondii*-infected cells release alarmin S100A11, which binds to the RAGE receptor on monocytes [59], yet no innate immune sensor has been shown to directly bind to *T*. *gondii* PAMPs. Our data suggest that an ITAM-bearing receptor or adaptor protein that activates Syk may serve as a potential recognition receptor, and this possibility is under investigation. We have previously identified a partial role for the parasite-secreted protein GRA15 in IL-1β production in primary human monocytes [35], and Syk signaling may synergize with GRA15 to induce maximal priming of IL-1β production.

Although IL-1β production was detected in all three subsets of monocytes by ICCS, in each donor examined, the CD14^+^CD16^-^ inflammatory monocytes produced more IL-1β in response to *T*. *gondii* than the other monocyte subsets. In human blood, this inflammatory subset of monocytes is present in significantly greater numbers than the other subsets. Recent research indicates that this CD14^+^CD16^-^ population of inflammatory monocytes is largely responsible for pathogenic inflammation in arthritis and sepsis [38,39], and our data are consistent with these findings. An intriguing possibility is that inflammatory monocytes regulate the expression or function of the receptors or signaling molecules involved in *T*. *gondii*-induced IL-1β production differently than the other monocyte subsets, rendering them more responsive to inflammatory stimuli.

Since the discovery that the fungal metabolite brefeldin A, which inhibits conventional protein secretion, did not inhibit IL-1β secretion from stimulated immune cells [22], the potential mechanisms of IL-1β release have been intensely studied. The best characterized mechanism of release occurs through an inflammatory form of cell death marked by cell swelling and lysis, termed pyroptosis [23]. Notably, IL-1β can also be released from viable cells in a pyroptosis-independent manner [29]. Indeed, this is the case with human, but not mouse, monocytes treated with LPS [21]. In 2015, the identification and characterization of GSDMD [24,25], which can be activated by the inflammasome and functions as the effector protein of pyroptosis by forming small pores in the cell membrane [26–28], provided a molecular basis for this inflammatory form of cell death. Interestingly, in the context of *T*. *gondii* infection, GSDMD cleavage and cell death did not appear to drive IL-1β release from primary human monocytes, as there was no difference in the percent of viable *T*. *gondii*-infected or mock-infected monocytes at 4 hpi, the time-point when functional IL-1β was detected in the supernatant. In addition, glycine treatment, which inhibits ion flux and pyroptosis, had no effect on *T*. *gondii*-induced IL-1β release. While it cannot be completely ruled out that a small number of monocytes that die early during *T*. *gondii* infection are responsible for all the IL-1β released, this possibility seems unlikely because *T*. *gondii* continues to live and replicate within human monocytes for at least another 14 hours after maximal IL-1β release is detected, suggesting that the cells do not die rapidly after infection. In examining a role for GSDMD, we found that the cleaved, active N-terminal fragment of GSDMD was not increased in *T*. *gondii*-infected primary monocyte lysates. Finally, GSDMD KO THP-1 cells released comparable levels of IL-1β to wild-type THP-1 cells during *T*. *gondii* infection, further suggesting a pyroptosis-independent mechanism of IL-1β release from *T*. *gondii*-infected monocytes.

Our current findings support and expand on a threshold model in which the amount of IL-1β production and the mechanism of its release are dependent on the stimulus [47]. The amount of IL-1β released by primary human monocytes during LPS stimulation was significantly higher than that released during *T*. *gondii* infection, and LPS and ATP stimulation together induced almost an order of magnitude more IL-1β release than LPS alone. Notably, only LPS and ATP stimulation triggered significantly more cell death than mock-treated monocytes. These data suggest that perhaps different signaling pathways are activated to induce low, medium, and high amounts of IL-1β release, all depending on the stimulus that a cell encounters. The use of a variety of stimuli that can lead to the same response, but perhaps through different mechanisms, will be a valuable tool in developing a more comprehensive understanding of how human immune cells regulate inflammation. This work also demonstrates that IL-1β production can be uncoupled from IL-1β release during LPS stimulation of primary human monocytes and highlights GSDMD-independent mechanisms of IL-1β release in the context of viable cells. Collectively, the current findings not only provide a more detailed understanding of how human innate immune cells regulate inflammation but also shed light on the pathways that contribute to host defense against a parasite pathogen of global importance.

## Materials and methods

### Ethics statement

Human whole blood was collected by the Institute for Clinical and Translational Science (ICTS) at the University of California, Irvine from healthy adult donors who provided written informed consent. Blood was collected according to the guidelines of and with approval from the University of California, Irvine Institutional Review Board (HS #2017–3753).

### Mammalian and *T*. *gondii* cell culture

Primary human monocytes were isolated from human whole blood collected by the Institute for Clinical and Translational Science (ICTS) at the University of California, Irvine from healthy adult donors. PBMCs were isolated from whole blood by density gradient centrifugation using lymphocyte separation media (MP Biomedicals, Santa Ana, CA). Monocytes were enriched from PBMCs by counterflow elutriation, as previously described [34], and stained for purity after isolation. This protocol typically resulted in >90% pure monocyte cultures (ranging from 85–95%) based on CD11b^+^ and CD3^−^CD20^−^CD56^−^ staining (S1 Fig). Freshly isolated monocytes were resuspended in RPMI 1640 (HyClone, Logan, UT) supplemented with 2 mM L-glutamine, 100 U/ml penicillin, 100 μg/ml streptomycin, and either 10% heat-inactivated FBS (Omega Scientific, Tarzana, CA) (R-10%) or no serum (R-0%). Monocytes were used immediately after isolation for experiments.

The human monocytic THP-1 cell line and the gasdermin D knock-out (GSDMD KO) THP-1 cells, a gift from Dr. Derek Abbott (Case Western Reserve University) [48], were cultured in R-10% (HyClone, Logan, UT) supplemented with 2 mM L-glutamine, 100 U/ml penicillin, and 100 μg/ml streptomycin. The Syk KO, CARD9 KO, and PKCδ KO THP-1 cells were cultured in R-10% (HyClone, Logan, UT) supplemented with 2 mM L-glutamine, 100 U/ml penicillin, 100 μg/ml streptomycin and 2 μg/ml puromycin.

Human foreskin fibroblasts (HFFs; from the lab of Dr. John Boothroyd, Stanford University School of Medicine) were cultured in D-10% medium: DMEM (HyClone) supplemented with 10% heat-inactivated FBS, 2 mM L-glutamine, 100 U/ml penicillin, and 100 μg/ml streptomycin. *T*. *gondii* tachyzoites were maintained by serial passage in confluent monolayers of HFFs. Type II (*Prugniaud*) [60] parasites constitutively expressing GFP were used.

All mammalian and parasite lines were cultured at 37°C in 5% CO_2_ incubators. All cultures were tested bimonthly and confirmed to be free of mycoplasma contamination.

### Generation of Knockout (KO) cell lines

Knockout THP-1 cells were generated using the Lenti-CRISPR-Cas9 system. Guide RNAs (sgRNA) targeting human Syk, PKCδ, or CARD9 were cloned into the LentiCRISPR v2 plasmid (Feng Zhang, Addgene plasmid #52961). Virus was generated by transfecting the sgRNA plasmid constructs into HEK 293T cells along with the psPAX2 packaging (Didier Trono, Addgene plasmid #12260) and pCMV-VSVG envelope (Bob Weinberg, Addgene plasmid #8454) plasmids. Viral supernatants collected 48 hr post-transfection were used to infect THP-1 cells by spinfection at 1800 rpm for 1 hr. Single-cell Syk knockout clones were generated by limiting dilution in 96-well plates under puromycin selection. Single-cell clones were sequenced after PCR amplification of a 500 bp region near the Cas9 binding site. Interference of CRISPR edits (ICE) analysis software (Synthego) was used to characterize the indel for each clone. A Syk KO clone (named 1–6) with a biallelic indel-induced frame-shift mutation in the second SH2 domain was used for subsequent experiments. The PKCδ and CARD9 KOs are comprised of mixed cell populations. All the mixed populations and clones were screened via Western blot for the presence of Cas9 and the absence of the gene targeted for deletion. The sequences for the guide RNAs were as follows: Syk: GAAAGAAGTTCGACACGCTC, PKCδ: AGTTCTTACCCACGTCCTCC, and CARD9: ATCGTTCTCGTAGTCCGACA.

### Infection and stimulation of monocytes

Primary human monocytes and monocytic THP-1 cells were resuspended in R-0% or R-10% medium directly after isolation and incubated with small molecule inhibitors or equal volumes of vehicle and incubated for 40 minutes at 37°C. *T*. *gondii*-infected HFF were washed with D-10% medium, scraped, and syringe lysed. Lysed tachyzoites were washed with R-0%, passed through a 5-μm filter (EMD Millipore, Billerica, MA), and washed with R-0% medium again. This resulted in parasite cultures that were free of host cell debris and soluble factors. Purified *T*. *gondii* tachyzoites were immediately added to host cells at a multiplicity of infection (MOI) of 2. All infections were performed with GFP-expressing type II *T*. *gondii*. “Mock” infections were samples in which an equivalent volume of culture medium without parasites was added to the cells.

Cells were stimulated with 100 ng/ml ultrapure *E*. *coli* LPS (List Biological Laboratories, Campbell, CA) and 5 mM ATP (Sigma-Aldrich, St. Louis, MO) for the last 30 min of culture, as indicated. “Mock” treatment was the addition of the equivalent volume of media (without parasites or LPS) to cells. At the indicated time point, monocytes were pelleted by centrifugation at 500 x g for 5 min. Collected cells were stained, fixed, or lysed accordingly, as described below.

### Inhibitors

MCC950 (Adipogen, San Diego, CA), glycine (Fisher Scientific, Waltham, MA) and potassium chloride (Fisher Scientific) were resuspended in deionized water. Go6983 (Selleck Chemicals, Houston, TX), Ac-Tyr-Val-Ala-Asp-chloromethylketone (Ac-YVAD-CMK or YVAD) (Cayman Chemical, Ann Arbor, MI), MI2 (Tocris Bioscience, Bristol, UK), PS1145 (Cayman Chemical), R406, and entospletinib (Selleck Chemicals), were all resuspended in DMSO. Monocytes were treated with the inhibitors or with an equivalent volume of the appropriate vehicle, for 40 min at 37°C and then infected or stimulated as described above. MCC950, Go6983, MI2, PS1145, R406 and entospletinib were all added to infected cells in half log titrations to determine the concentrations of the inhibitors that did not induce cell death or reduce infection efficiency.

### Plaque assays

HFF were plated on 6-well plates and grown to confluence for two days. HFF were pretreated with specific inhibitors for 40 min and then infected with freshly-lysed *T*. *gondii* tachyzoites for 5–7 days, followed by fixation with 10% Neutral Buffered Formalin. Staining was done using 600 μg/ml of Neutral Red solution overnight. The plaques were manually counted and imaged using a Leica DMi8 microscope with a DMC 5400 camera.

### Flow cytometry

To measure infection efficiency, cells were harvested at the time points listed and immediately analyzed by FACS to determine the percent of GFP^+^ (*T*. *gondii*-infected) cells. To measure cell viability, cells were harvested, washed and resuspended in FACS buffer (2% FBS in PBS) containing propidium iodide and analyzed by flow cytometry without fixation. For cell surface staining, cells were blocked with Human TruStain FcX (BioLegend, San Diego, CA) on ice for 10 min and then stained with control Ig or the following anti-human Abs (all from BioLegend, unless otherwise indicated): anti-CD56–allophycocyanin (HCD56), anti-CD11b–PE or, anti-CD14–FITC (M5E2) or -PE/Cy7 (HCD14), anti-CD16-PE/Cy7 or -APC (3G8), anti-CD3–PE (UCHT1), or anti-CD20–PE/Cy7 (2H7). Cells were stained with the Abs on ice for 30 min, washed with FACS buffer, and either run live or fixed with 2–4% paraformaldehyde. For intracellular cytokine staining (ICCS), cells were fixed and permeabilized with 100 μL of BD Cytofix/Cytoperm solution (BD Biosciences, Franklin Lakes, NJ) for 20 minutes on ice. After incubation, cells were washed with FACS buffer containing 0.1% Triton-X, blocked with Human TruStain FcX as described above, stained with control Ig-PE or anti-IL-1β–PE (CRM56; eBioscience, San Diego, CA), control Ig-PE/Cy7 or anti-phospho-Syk (Y525/526)-PE-Cy7 (C87C1; Cell Signaling Technologies, Danvers, MA) Abs for 30 min, and washed with FACS buffer.

Samples were analyzed by flow cytometry on a FACSCalibur flow cytometer using CellQuest software (BD Biosciences). Data were analyzed using FlowJo software (TreeStar, Ashland, OR). Cells were first identified based on their forward and side scatter profile and subsequently analyzed for cell surface marker expression, intracellular cytokine expression, or GFP signal.

### Quantitative real-time PCR (qPCR)

At the harvest time point, total RNA was harvested using the RNeasy Kit (QIAGEN, Germantown, MD) and treated with DNase I (Life Technologies, Carlsbad, CA) to remove any contaminating genomic DNA. cDNA was synthesized using the Superscript III First-Strand Synthesis kit (Life Technologies), according to the manufacturer’s instructions, and subsequently used as template in quantitative real-time PCR (qPCR). qPCR was performed in triplicate using a Bio-Rad iCycler PCR system (Bio-Rad, Hercules, CA) and iTaq Universal SYBR Green Supermix (Bio-Rad). Previously published sequences for *IL-1β*, *NLRP3* and *GAPDH* primers were used [34]. All primer pairs spanned intron-exon boundaries whenever possible and bound to all isoforms of the gene, where applicable. All primers were commercially synthesized by Integrated DNA Technologies (Coralville, IA). qPCR data were analyzed using the threshold cycle method, as previously described [34], and gene expression data are shown normalized to that of the housekeeping gene GAPDH. In all qPCR assays, cDNA generated in the absence of reverse transcriptase, as well as water in the place of DNA template, were used as negative controls, and these samples were confirmed to have no amplification.

### ELISA

Human IL-1β protein released into the supernatant was measured using ELISA MAX Deluxe kits (BioLegend), according to the manufacturer’s instructions. Signal from ELISA plates was read with a Spectra Max Plus 384 plate reader (molecular Devices, San Jose, CA) using SoftMax Pro Version 5 software (molecular Devices), and the threshold of detection was 0.5 pg/ml.

### Western blots

At the harvest time point, cells were lysed by addition of 2X Laemmli buffer containing 10% 2-ME. For experiments in which supernatant was analyzed, serum-free R-0% medium was used during the infection, and supernatant was concentrated using Amicon Ultra Centrifugal filters (EMD Millipore, Burlington, MA), according to the manufacturer’s instructions. Concentrated supernatant was diluted with 2X Laemmli buffer containing 10% 2-ME. Samples were boiled at 100°C for 10 to 15 min, separated by SDS-PAGE, and transferred to polyvinylidene difluoride (PVDF) membranes (Bio-Rad, Hercules, CA) for immunoblotting. Membranes were blocked for 1 h at room temperature (RT) with blocking buffer: 5% non-fat milk or 5% bovine serum albumin (BSA) (Fisher Bioreagents). Membranes were then incubated with primary antibodies diluted in blocking buffer for 1 h at RT or overnight at 4°C. Membranes were probed with antibodies against NF-κB p65 (D14E12; Cell Signaling), phospho-NF-κB p65 (Ser536) (93H1; Cell Signaling), total Syk (2712S; Cell Signaling), phospho(Tyr525/526)-Syk (2711S; Cell Signaling), gasdermin D (NPB2-33422; Novus Biologicals, Littleton,CO), or β-actin (AC-15; Sigma-Aldrich). Membranes were blotted for IL-1β (3ZD from the National Cancer Institute Biological Resources Branch) using the SNAP i.d. Protein Detection System (EMD Millipore), according to the manufacturer’s instructions. Primary Abs were followed by HRP-conjugated secondary Abs (BioLegend), and membranes were developed with SuperSignal West Femto Maximum Sensitivity Substrate (Thermo Fisher Scientific, Carlsbad, CA), Amersham ECL Prime Western Blotting Detection Reagent (GE Healthcare, Little Chalfont, U.K.) substrate or ECL Prime Substrate (Thermo Fisher Scientific). Signal was detected using a Nikon camera, as previously described [34]. Quantification analysis of blots was performed using ImageJ software.

### HEK-Blue reporter cell assay

HEK-Blue IL-1 reporter cells (Invivogen, San Diego, CA), which respond to IL-1 binding to the IL-1R, were incubated in D-10% medium supplemented with 2 mM L-glutamine, 100 U/ml penicillin, 100 ug/ml streptomycin, Normocin (100 μg/ml), Hygromycin B Gold (200 μg/ml) and Zeocin (100 μg/ml). For the detection of IL-1 released from THP-1 cells and primary human monocytes, HEK-Blue cells were resuspended at a concentration of 500,000 cells/ml and added to flat-bottom 96-well plates. Supernatants collected from THP-1 cells or primary monocytes were added to the HEK-Blue cells and incubated for 24 hours at 37°C. The HEK-Blue cell supernatant was combined with the QUANTI-Blue detection reagent (Invivogen), incubated at 37°C for 1 to 3 hours, and then quantified with a Spectra Max Plus 384 plate reader (Molecular Devices, San Jose CA) using SoftMax Pro Version 5 (Molecular Devices) software.

### Statistics

Statistical analyses were performed using GraphPad Instat software. Analysis of variance (ANOVA) followed by Tukey’s or Bonferroni’s test, as indicated, were used for comparison between means. Differences were considered significant when the *P* value was <0.05.

## Supporting information

S1 Fig Phenotype of primary human monocytes. Primary monocytes were enriched from PBMCs of healthy blood donors by counterflow elutriation and analyzed for purity. Cells were stained with anti-CD56, anti-CD11b, anti-CD20, anti-CD3 or isotype controls (cIg) for each antibody, and flow cytometry was performed. The results of a representative analysis from > 70 independent monocyte isolation experiments are shown.
(EPS)

S2 Fig Effect of inhibitors on infection efficiency and parasite viability. (**A**) Primary human monocytes were pretreated with 50 mM potassium chloride, 2 μM MCC950, 50 μM YVAD, 0.3 μm entospletinib, 300 nM Go6983, 3 μM MI2, 100 nM PS1145, or vehicle control for 40 min. Cells were then infected with *T*. *gondii* for 4 h, and infection efficiency (% of GFP+ cells) was analyzed by flow cytometry. (**B**) HFFs were grown in 6-well plates and pre-treated with 2 μM MCC950, 0.3 μm entospletinib, 300 nM Go6983, 3 μM MI2, 100 nM PS1145, or vehicle control for 40 min before infection with *T*. *gondii*. Plaque assays were conducted and plaques in each condition were counted. For (A), representative plots from 2–9 independent donors are shown. 1 representative plaque assay of 3 independent experiments is shown in (B).
(EPS)

S3 Fig Effect of the Syk-specific inhibitor R406 on infection efficiency and parasite viability. (**A**) Primary human monocytes were pretreated with 2 μM R406 or vehicle control for 40 min and infected with *T*. *gondii* for 4 h or 16 h. The infection efficiency (% of GFP+ cells) and the median fluorescence intensity (MFI) of the GFP^+^ population was analyzed by flow cytometry. (**B**) HFFs were grown in 6-well plates and pre-treated with a titration of R406 or vehicle control for 40 min before infection with *T*. *gondii*. Plaque assays were conducted, and plaques in each condition were counted. (**C**) Primary human monocytes were pretreated with a vehicle control or R406 and either infected with *T*. *gondii*, stimulated with LPS alone, or LPS+ATP, and stained with propidium iodide. The cells were analyzed by flow cytometry for cell viability. These data show the representative FACS plots for the graph shown in Fig 7A. For (A), a representative set of FACS plots is shown, and the GFP MFI and infection efficiency graphs reflect data from 3 independent experiments. 1 representative plaque assay of 2 experiments is shown in (B). Values are expressed as the mean ± SD, ***P<0.001, (one-way ANOVA followed by a Tukey post-test in panel A).
(EPS)

S4 Fig Generation of Syk KO clone in THP-1 cells. (**A**) THP-1 cells were mock treated or infected with *T*. *gondii* for 30 min. Total Syk, phospho-Syk (Tyr 525/526), and β-actin in the cell lysates were visualized by Western blotting. (**B**) Syk KO clone 1–6 contains an indel in both alleles (biallelic indel) that introduces a frameshift mutation in the second SH2 domain. The wild-type amino acid (aa) sequence of Syk near the Cas9 binding site is shown above, and the aa sequences of the two alleles in the KO clone are shown below, with the mutated sequences shown in red. (**C**) Interference of CRISPR edits (ICE) software analysis of Syk clone 1–6 generated an indel frequency plot (left) showing the relative frequency of each indel based on their number of nucleotides (indel sizes), with approximately equal frequencies of the two indels for the biallelic KO clone. Discordance plots (right) show the alignment of bases between the wild-type unedited sequence (red) and the KO sequence (green), with discordance observed near the Cas9 cut site. Vertical dotted lines denote the expected cut site.
(EPS)

S5 Fig ATP triggers cell death in a dose-dependent manner. Primary monocytes were stimulated with LPS (100 ng/ml) alone or in combination with ATP (0.3, 1.0, or 5.0 mM), or vehicle control for 4 h, and stained with propidium iodide (PI). Cell viability was analyzed by flow cytometry. Values are expressed as the mean ± SD from experiments with n = 3 independent donors. *P<0.05, ***P<0.001 (one-way ANOVA followed by a Tukey post-test).
(EPS)
